# Endoscopic ultrasound-guided biliary recanalization with a novel rendezvous inflated balloon-assisted technique for isolated bile leakage

**DOI:** 10.1055/a-2098-1562

**Published:** 2023-06-15

**Authors:** Naruomi Jinno, Michihiro Yoshida, Kazuki Hayashi, Itaru Naitoh, Yasuki Hori, Makoto Natsume, Hiromi Kataoka

**Affiliations:** 1 Department of Gastroenterology and Metabolism, Nagoya City University Graduate School of Medical Sciences, Nagoya, Japan; 2 Department of Gastroenterology, Japan Community Health Care Organization Chukyo Hospital, Nagoya, Japan; 3 Department of Gastroenterology, Nagoya City University East Medical Center, Nagoya, Japan

Bile leakage after hepatectomy has been reported to occur in 5 %–8 % of cases 1 2 . In particular, isolated bile leakage is intractable and may require surgical re-anastomosis. In general, endoscopic treatment for isolated bile leakage by transpapillary biliary drainage for recanalization is challenging 3 ; the procedure is often unsuccessful because of surgically altered anatomy and disconnection of the bile duct. Here, we report a case of successful endoscopic ultrasound (EUS)-guided biliary recanalization for isolated bile leakage that employed a novel approach assisted by rendezvous balloon inflation.

A 74-year-old man with gallbladder cancer underwent cholecystectomy with partial hepatectomy and bile duct resection. Following the surgery, isolated bile leakage occurred at the right posterior branch (RPB) ( Fig. 1 ). Initially, percutaneous transhepatic biliary drainage (PTBD) of the RPB was attempted; however, percutaneous guidewire negotiation across the obstructed duct failed because of the complete disconnection. We then performed EUS-guided biliary drainage (EUS-BD) to create internal drainage ( Video 1 ). Investigation of the RPB by EUS failed because of nondilation of the RPB. We introduced a 6-mm dilation balloon catheter via the PTBD route and inflated the balloon, which served as a target for EUS-guided needle puncture ( Fig. 2 a, b ). The inflated balloon was successfully punctured, and a guidewire was inserted under EUS guidance and grasped with biopsy forceps (SpyBite; Boston Scientific, Natick, Massachusetts, USA) under direct cholangioscopic visualization (SpyGlass DS; Boston Scientific) via the percutaneous rendezvous approach. Finally, we were able to advance a 10.2-Fr catheter into the duodenum along the guidewire and achieve successful internal drainage ( Fig. 3 ).

**Fig. 1:**
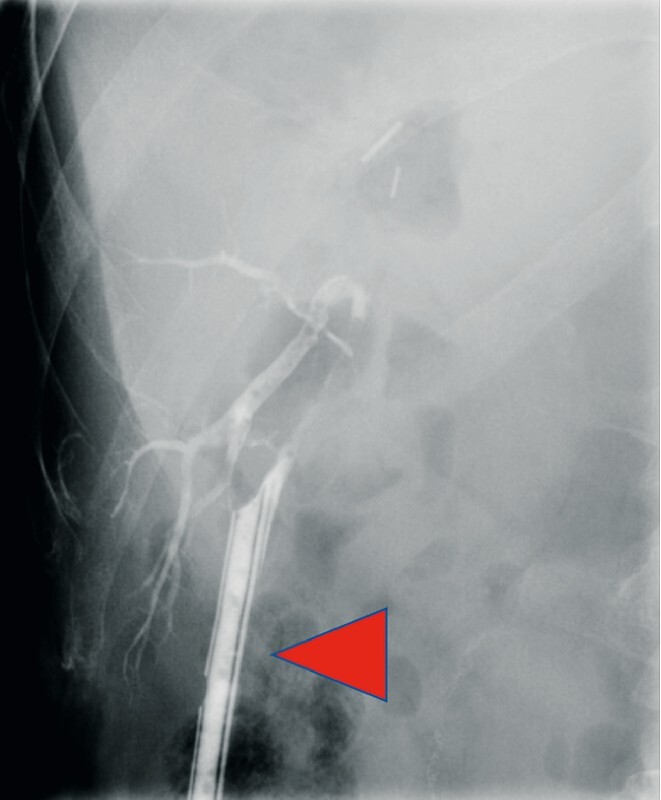
Cholangiography performed after surgery through the inserted intra-abdominal drainage tube (red triangle) outlined only the right posterior branch (RPB) and indicated isolated bile leakage at the RPB.

**Video 1** Successful endoscopic ultrasound-guided biliary recanalization with rendezvous balloon-inflation assistance and cholangioscopy to manage isolated bile leakage.


**Fig. 2:**
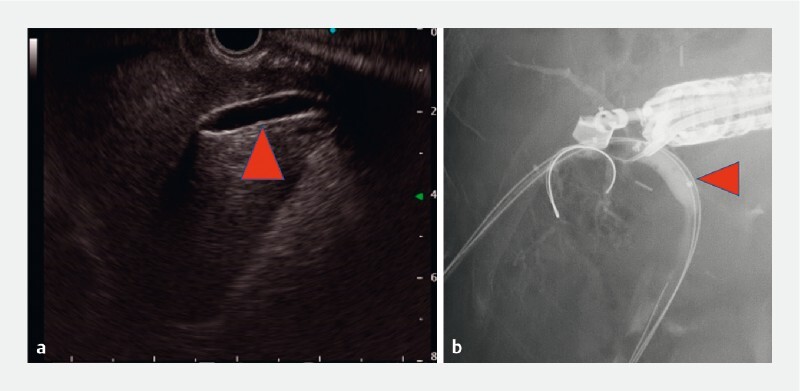
A guidewire was successfully inserted into the right posterior branch by: **a** inserting a dilation balloon catheter via the percutaneous transhepatic biliary drainage (PTBD) route, with the inflated balloon (red triangle) scanned using endoscopic ultrasound (EUS), then; **b** puncturing the inflated balloon with an EUS-guided needle, to enable insertion of the guidewire.

**Fig. 3:**
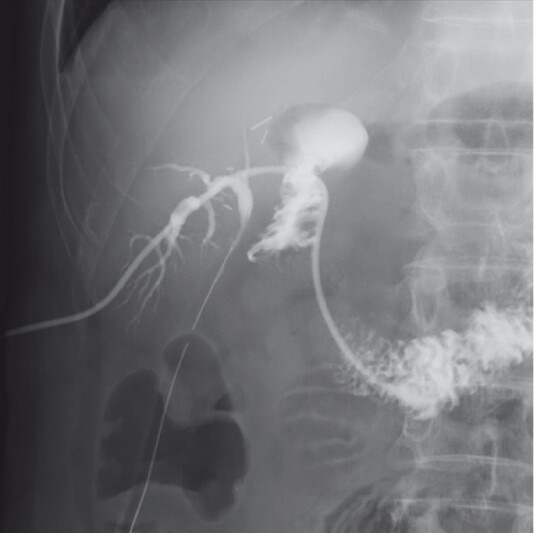
Duodenography via the percutaneous transhepatic biliary drainage (PTBD) route showing internal drainage successfully achieved via the rendezvous approach after insertion of a cholangioscope from the PTBD route allowed the guidewire coming from the endoscopic ultrasound-guided biliary drainage route to be grasped with biopsy forceps, so that a plastic stent could be deployed via the percutaneous route into the duodenum along the guidewire.

Rendezvous cholangioscopic assistance has been reported to be a useful technique for successful recanalization of postoperative biliary disconnection 4 . In addition, EUS-guided drainage of an open pancreaticocutaneous fistula using the balloon as a target has been reported 5 . Rendezvous balloon-inflation assistance is also a useful option for EUS-BD in the treatment of refractory isolated bile leakage.

Endoscopy_UCTN_Code_TTT_1AS_2AD

## Notes

Endoscopy E-Videos

https://eref.thieme.de/e-videos
*E-Videos* is an open access online section of the journal *Endoscopy* , reporting on interesting cases and new techniques in gastroenterological endoscopy. All papers include a high-quality video and are published with a Creative Commons CC-BY license. Endoscopy E-Videos qualify for HINARI discounts and waivers and eligibility is automatically checked during the submission process. We grant 100% waivers to articles whose corresponding authors are based in Group A countries and 50% waivers to those who are based in Group B countries as classified by Research4Life (see: https://www.research4life.org/access/eligibility/ ). This section has its own submission website at https://mc.manuscriptcentral.com/e-videos
